# Effects of aging on hydrocephalus after intraventricular hemorrhage

**DOI:** 10.1186/s12987-020-0169-y

**Published:** 2020-02-28

**Authors:** Yingfeng Wan, Feng Gao, Fenghui Ye, Weiming Yang, Ya Hua, Richard F. Keep, Guohua Xi

**Affiliations:** 1 Department of Neurosurgery, University of Michigan, R5018 Biomedical Science Research Building, 109 Zina Pitcher Place, Ann Arbor, MI 48109-2200 USA; 2 Department of Neurosurgery, Sir Run Run Shaw Hospital, Zhejiang University, Hangzhou, China; 3 Department of Neurology, 2nd Affiliated Hospital, Zhejiang University, Hangzhou, China

**Keywords:** Aging, Intraventricular hemorrhage, Hydrocephalus, Heme oxygenase-1, Epiplexus cells, Marcrophages, Iron

## Abstract

**Background:**

Hydrocephalus is a common and major complication that affects outcome after intraventricular hemorrhage (IVH). While aging impacts the occurrence of hydrocephalus in patients with IVH this and the underlying mechanisms have received little attention. The present investigation, therefore, studied the impact of aging on hydrocephalus after IVH in a rat model.

**Methods:**

Young and aged (3 and 18 months old, respectively) male Fischer 344 rats had an intraventricular injection of 200 μl autologous blood or saline. Ventricular volume was estimated using magnetic resonance imaging (MRI), while ventricular wall damage, heme oxygenase-1 (HO-1) and epiplexus cell activation were quantified by histological staining and Western blot. Additionally, the impact of intraventricular iron injection was examined in young and aged rats.

**Results:**

Intraventricular injection of autologous blood induced hydrocephalus in both young and aged rats but ventricular volumes were larger in aged rats compared to young rats from day 3 to day 14 followed IVH. In addition, ventricular wall damage and periventricular HO-1 upregulation were greater in aged versus young rats on day 1 after IVH. Aged rats also had more choroid plexus epiplexus cells on day 14 after IVH. Additionally, organized hematomas were observed in 23% (3/13) of aged rats but not in young rats after IVH. Organized hematomas in aged rats showed larger T2* lesions on MRI compared to rats with non-organized hematomas. Similar to the effects of IVH, intraventricular injection of iron resulted in more epiplexus cells activation and more severe hydrocephalus in aged compared to young rats.

**Conclusions:**

IVH causes more severe hydrocephalus in aged compared to young rats. Enhanced ventricular wall damage, epiplexus cell activation and iron overload may contribute to this aggravated hydrocephalus development in aged animals.

## Background

In adults, intraventricular hemorrhage (IVH) secondary to intracerebral hemorrhage (ICH) or subarachnoid hemorrhage is linked to high morbidity and mortality. For example, secondary IVH after intracerebral hemorrhage (ICH) in adults is correlated with worse prognosis [1, 2] being associated with impaired consciousness at presentation as well as long-term functional impairment [2]. IVH can cause post-hemorrhagic hydrocephalus, a major cause of neurological disability that can create a lifelong dependence on medical care. IVH is also a common disease in premature neonates. In the United States, over 12,000 infants develop hydrocephalus after IVH per year [3].

Many patients that have IVH after ICH are elderly. For example, in the Surgical Trial in Intracerebral Hemorrhage (STICH) Trial [1], the mean age was 60.9 years in patients with secondary IVH following ICH. Importantly, 58% of the patients greater than or equal to 50-year-old developed hydrocephalus after IVH, compared to only 41% of patients less than 50-year-old. Our previous study found that ICH-induced brain injury and neurological deficits were greater in aged animals [4]. However, the mechanisms by which aging might impact IVH-induced brain injury and particularly hydrocephalus development are still unclear.

Multiple mechanisms have been implicated for IVH-induced hydrocephalus including altered CSF absorption, damage to ventricular wall and periventricular tissue, inflammation and iron-overload due to erythrocyte lysis. Thus, much research has suggested that ependymal damage may lead to hydrocephalus development [5–7]. Inflammation plays a role in hydrocephalus development after subarachnoid hemorrhage [8] and recent research indicates choroid plexus inflammatory signals can impact cerebrospinal fluid (CSF) secretion and hydrocephalus development in rat IVH [9]. We also found that spontaneously hypertensive rats exhibited epiplexus cell activation during the hydrocephalus that normally occurs during development in that strain [10]. Epiplexus cells (also known as Kolmer cells) are macrophages residing on the apical surface of the choroid plexus. Iron released from hemoglobin after RBC lysis plays a crucial part in the brain damage after ICH [11] and intraventricular iron can induce hydrocephalus in rats [12]. One potentially important player in iron toxicity is heme oxygenase-1 (HO-1) that degrades hemoglobin resulting in the release of iron. It is highly upregulated in the periventricular zone after IVH, particularly in microglial cells [5]. The impact of aging on these types of injury/response after IVH is uncertain.

The purpose of the current study was, therefore, to determine whether the degree of IVH-induced hydrocephalus differed between young and aged rats. It also investigated whether age impacted ependymal damage, periventricular HO-1 expression and epiplexus cell activation that might correlate with differences in hydrocephalus development. Finally, it examined a potential underlying mechanism, differences in iron toxicity between young and aged rats.

## Methods

### Animal preparation and intraventricular injection

The animal use protocols were approved by the Committee on the Use and Care of Animals of the University of Michigan. Forty-six 3-month-old and forty-eight 18-month-old male Fischer 344 rats (National Institutes of Health, Bethesda, MD), were utilized in this research. Henceforth, these groups are defined as young and aged rats, respectively. Rats were anesthetized with pentobarbital (50 mg/kg, intraperitoneally) and body temperature was maintained at 37 °C using a heating blanket. A polyethylene catheter was used to cannulate the right femoral artery for collection of autologous arterial blood and monitoring blood pressure, arterial blood pH, arterial PaO_2_ and PaCO_2_, hematocrit and blood glucose. A stereotaxic frame (Kopf Instruments, Tujunga, CA) was used to position the animal and a 26-gauge needle was inserted into the right lateral ventricle (coordinates: 0.6 mm posterior, 4.5 mm ventral, and 1.6 mm lateral to the bregma) through a drilled cranial hole (1 mm), following by an infusion of 200 μl autologous arterial blood or saline (14 μl/min) with a microinfusion pump (World Precision Instruments Inc., Sarasota, FL). After the removal of the injection needle, the burr hole was filled with bone wax, the skin sutured and the animal allowed to recover.

### Experimental groups

The study was separated into three parts. In the first part, 200 μl of autologous arterial blood or saline was injected into the right lateral ventricle of both young and aged rats. The animals underwent magnetic resonance imaging (MRI) and were euthanized on day 1 after intraventricular infusion. The brains were harvested for Western blotting (n = 4 for each group) and histological staining (n = 6 for each group). In the second part, 200 µl of autologous arterial blood or saline (pH 6.9) was injected into the right lateral ventricle of both young and aged rats, and serial MRIs were carried out on day 1, 3, 7 and 14. Rats were then euthanized on day 14 and the brains harvested for histological staining (n = 13 for aged IVH and young IVH groups, n = 6 for aged control and young control groups). In the third part, FeCl_3_ (2 mmol/L, pH 3.0, 50 µl) or saline was injected into the right lateral ventricle of both young and aged rats over 5 min. The animals went through MRI scanning and were euthanized one day after intraventricular infusion. Brains were harvested for histology (n = 7 for each group).

### MRI and ventricle volume estimation

Rats were anesthetized using ~ 2% isoflurane during the MRIs. T2 fast spin-echo sequence (TR/TE = 4000/60 ms) and a T2* gradient-echo (GRE) sequence (TR/TE = 250/5 ms) were performed with a 7.0-T Varian MR scanner (Varian Inc.) The field of view was 35 mm × 35 mm, and the matrix was 256 × 128 mm. A total of 25 coronal slices (0.5 mm thick) were acquired in each sequence to cover the entire lateral ventricles. Bilateral ventricular volumes calculation was performed as described previously [5]. Ventricular volume was obtained by multiplying ventricle areas of all slices and the section thickness. Images were analyzed with Image J software (National Institutes of Health, Bethesda, MD) by a blinded investigator.

### Ventricular wall damage analysis

Ventricular wall damage is presented as  % ependymal damage as described previously [6]. In brief, brain coronal sections with hematoxylin and eosin (H&E) staining were used and the length of ependymal cell discontinuities and detachments were measured and divided by the total ventricular perimeter. Images were analyzed with Image J software by a blinded investigator.

### Immunohistochemistry and immunofluorescence staining

Rats were euthanized using pentobarbital (100 mg/kg, intraperitoneal) and perfused intravascularly with 4% paraformaldehyde in 0.1 mol/L phosphate-buffered saline (pH 7.4). Brains were harvested and sectioned into 18-μm-thick slices with a cryostat after embedding. Immunohistochemical and immunofluorescence studies were performed as previously described [13]. The primary antibodies were rabbit anti-HO-1 (1:400 dilution; Abcam, Cambridge, USA), goat anti-Iba-1 (1:400 dilution; Abcam), mouse anti-CD68 (1:100 dilution; Abcam), mouse anti-rat CD163(1:100 dilution; AbD Serotec, Hercules, USA), polyclonal rabbit anti-alpha smooth muscle actin (1:200 dilution; Abcam). The secondary antibody in the immunofluorescence studies was Alexa Fluor 594 donkey anti-rabbit IgG (1:500, Invitrogen, Carlsbad, USA). Nuclear labeling was performed using fluoroshield™ with DAPI (F6057). Negative controls were performed without primary antibodies.

### Cell counting

Histological staining of sections at approximately − 3.8 mm from bregma was observed under a microscope and pictures were captured by a digital camera. Calculation of immuno-positive cells was performed on 3 separate images (×40 magnification) in each section in the periventricular area. The percentage of Iba1 and CD68 immune-positive macrophages was calculated using the number of immune-positive cells divided by the total number of choroidal epithelial cells in the same brain section. All analyses were performed using the Image J software by an investigator who was blinded to animal information, and the mean value of three repeat analyses was used.

### Western blot analysis

Western blotting was conducted as previously described [14]. Briefly, periventricular brain tissue (~ 1 mm thick around the ventricles) was sampled and sonicated in Western sample buffer. Bio-Rad protein assay kit was used to equalize the protein amount in each sample. Samples were then separated with a sodium dodecyl sulfate-polyacrylamide gel electrophoresis and transferred to a Hybond-C pure nitrocellulose membrane (Amersham, Pittsburgh, USA). The primary antibody was rabbit anti-HO-1 (1:2000 dilution; Abcam). To visualize the antigen–antibody complex, the ECL chemiluminescence system (Amersham) and a Kodak X-OMAT film were used. The image was analyzed by Image J software to determine relative densities.

### Statistical analysis

Results are presented as mean ± standard deviation (SD) and analyzed by Student *t* test or one-way ANOVA with a Tukey’s post hoc test. Differences were considered significant at *p *< 0.05.

## Results

The mortality rate was 8% (2/25) after intraventricular injection of 200 µl autologous arterial blood in aged (18 months-old) rats. None of the aged rats with intraventricular saline (n = 16) or iron (n = 7) injection died, nor did any of the young (3 months-old) rats (n = 39). Physiological parameters of some rats were monitored during intraventricular infusions. No difference in the mean arterial blood pressure, blood pH, arterial blood gases, hematocrit, and blood glucose was found between groups. There was a difference in body weight between young rats and aged rats (Table 1).

**Table 1:** Physiological parameters

	n	Weight (g)	MABP (mm Hg)	pH	pO_2_ (mm Hg)	pCO_2_ (mm Hg)	Hematocrit (%)	Glucose (mg/dl)
Aged IVH	6	452 ± 30.4^##^	116 ± 11.5	7.42 ± 0.03	83.8 ± 6.3	45.0 ± 4.3	42.8 ± 3.9	127 ± 15.8
Young IVH	6	255 ± 9.0	104 ± 10.5	7.40 ± 0.03	82.3 ± 7.4	44.3 ± 2.6	42.2 ± 3.2	116 ± 13.4
Aged control	6	463 ± 23.8^##^	115 ± 10.8	7.41 ± 0.03	83.0 ± 6.7	44.5 ± 3.7	42.3 ± 3.4	124 ± 16.2
Young control	6	265 ± 9.8	105 ± 12.0	7.41 ± 0.03	82.8 ± 6.4	43.5 ± 3.1	41.7 ± 2.7	118 ± 19.1

Values are expressed as the mean ± SD. ^##^p < 0.01 vs young rats

Young: 3 months old, Aged: 18 months old, IVH: intraventricular hemorrhage, MABP: mean arterial blood pressure

### Ventricle enlargement in young and aged rats after IVH

**Fig. 1:**
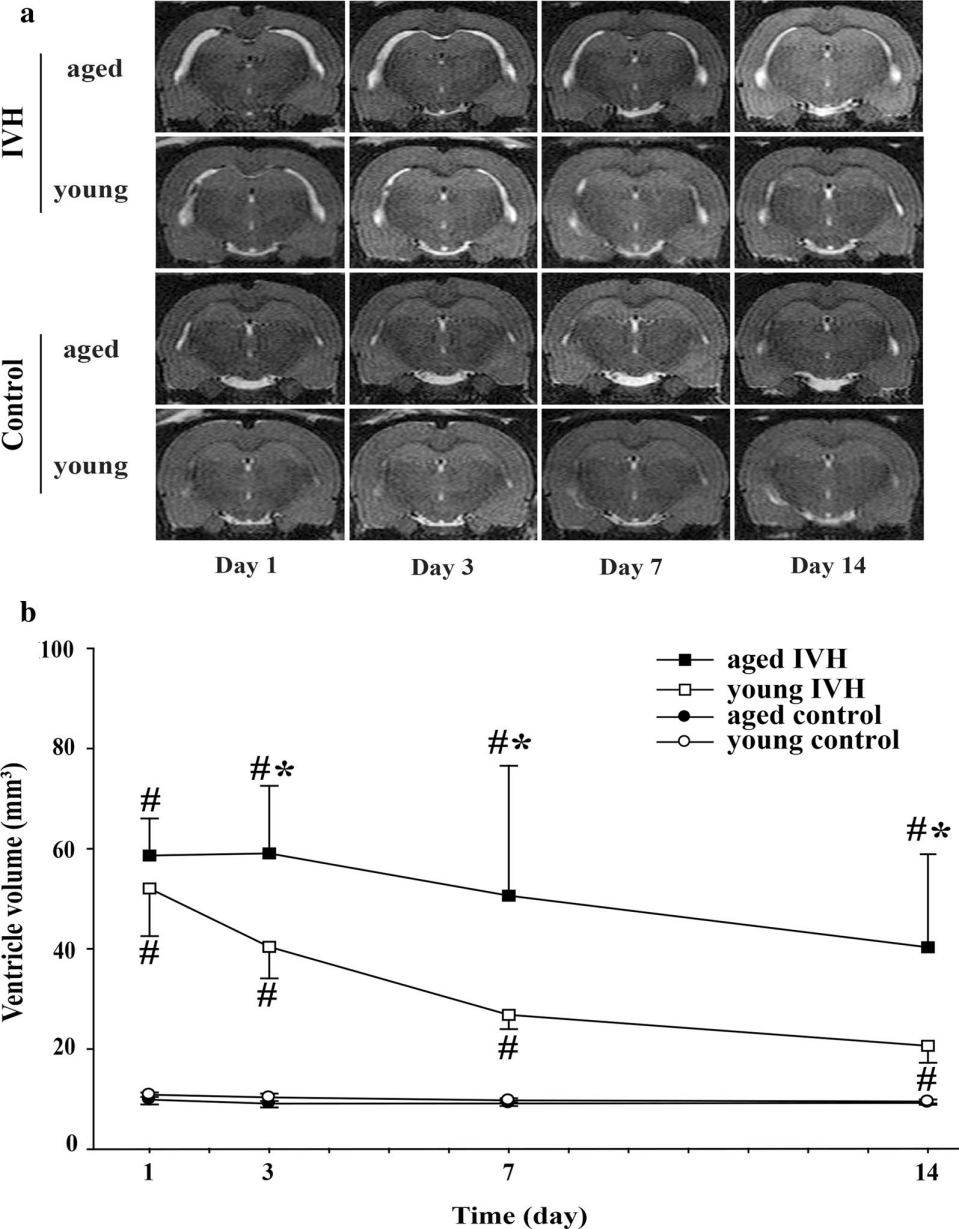
**a** Examples of T2-weighted MRI scans at day 1, 3, 7 and 14 after intraventricular injection of blood (200 µl) or saline in young (3 months) and aged (18 months) F344 rats. Note the dilated ventricles in the blood injected rats. **b** Ventricular volume was quantified using T2-weighted MRI scans in the young and aged F344 rats. Values are mean ± SD; n = 13 in aged intraventricular hemorrhage (IVH) group and n = 6 in other three groups. #p< 0.01 IVH vs. respective control groups, *p < 0.01 aged IVH vs. young IVH group by one-way ANOVA

Intraventricular injection of 200 µl autologous arterial blood resulted in bilateral ventricles enlargement of both young and aged animal in serial MRI scanning (Fig. 1a). The ventricular volumes were significantly larger in both young and aged IVH rats than the ventricular volumes in control rats from day 1 to 14 (p < 0.01, Fig. 1b). The ventricular volumes showed no difference between aged IVH and young IVH rats on day 1 (58.6 ± 7.4 vs. 52.1 ± 9.5 mm^3^ in the young rats, p > 0.05, Fig. 1b). However, the ventricular volumes of aged IVH rats were larger than those of young IVH rats from day 3 to 14 (p < 0.01, Fig. 1b). Young and aged control rats had similar ventricular volumes during the observation period (p > 0.05, Fig. 1b).

### Ependymal damage and HO-1 levels in young and aged rats after IVH

**Fig. 2:**
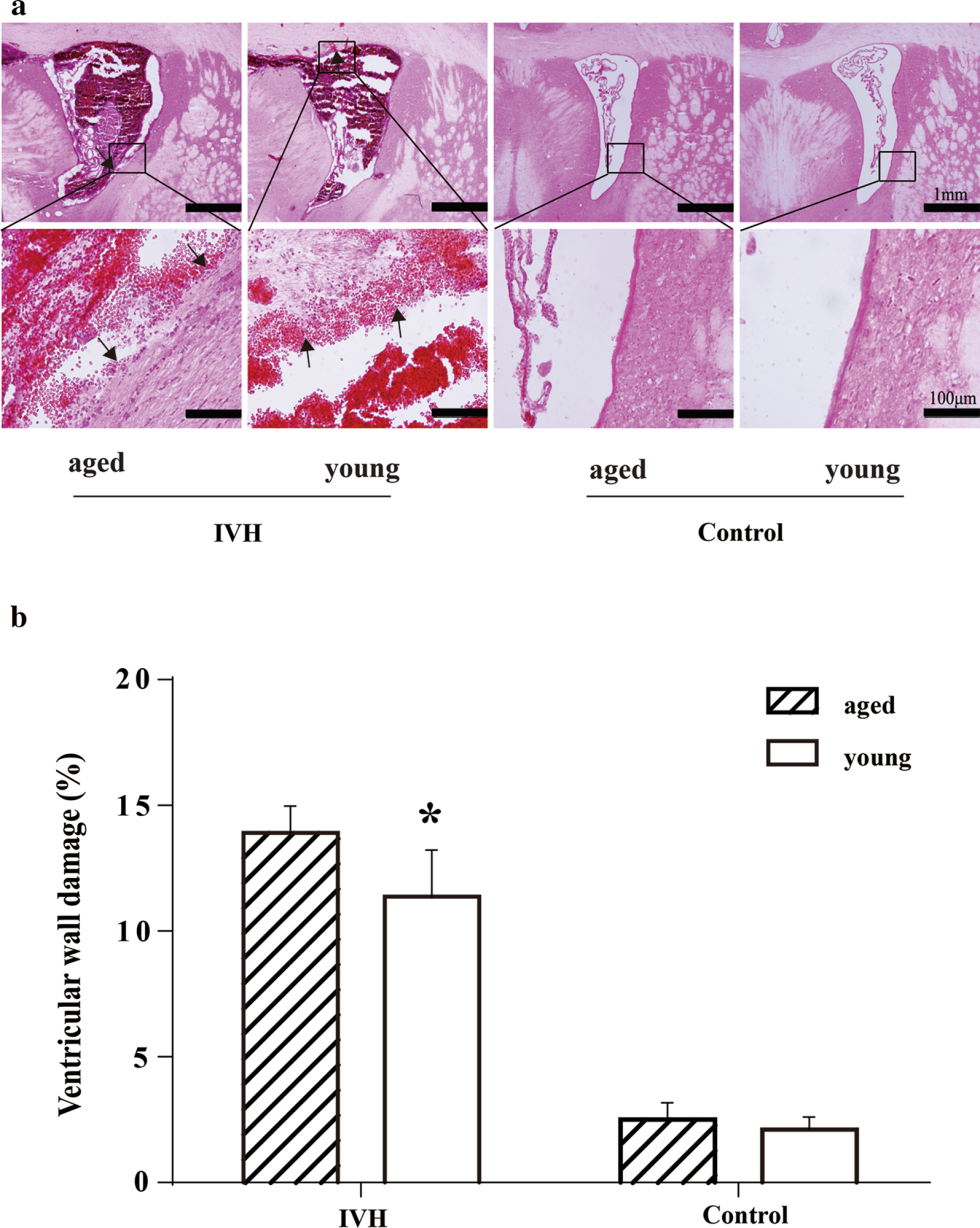
**a** Hematoxylin and eosin staining showing ventricle wall disruption one day after intraventricular injection of blood (200 µl) or saline in the young (3 months) and aged (18 months) F344 rats. Scale bar = 1 mm (upper row) and 100 µm (lower row). **b** The percentage of the ventricular wall that was damaged was determined for each animal (bar graph). Values are mean ± SD, n = 6, *p < 0.05 vs. young IVH group by Student *t* test

To evaluate differences in initial damage after IVH between the young and aged rats, animals were euthanized on day 1 following intraventricular injection of 200 µl autologous arterial blood or saline. The ependymal cells of the ventricular wall showed noticeable damage in both young and aged IVH rats but not in control groups (H&E staining, Fig. 2a). The percentage of ventricular wall damage (break down of the ependymal layer) was elevated on day 1 in both young and aged IVH groups compared with control groups, but was higher in aged rats (13.9 ± 1.1 vs. 11.4 ± 1.9% in the young rats, p < 0.05, Fig. 2b). No difference in ventricular wall integrity disturbance was found between young and aged control groups (2.5 ± 0.7 vs. 2.1 ± 0.5% in the young rats, p > 0.05, Fig. 2b).

**Fig. 3:**
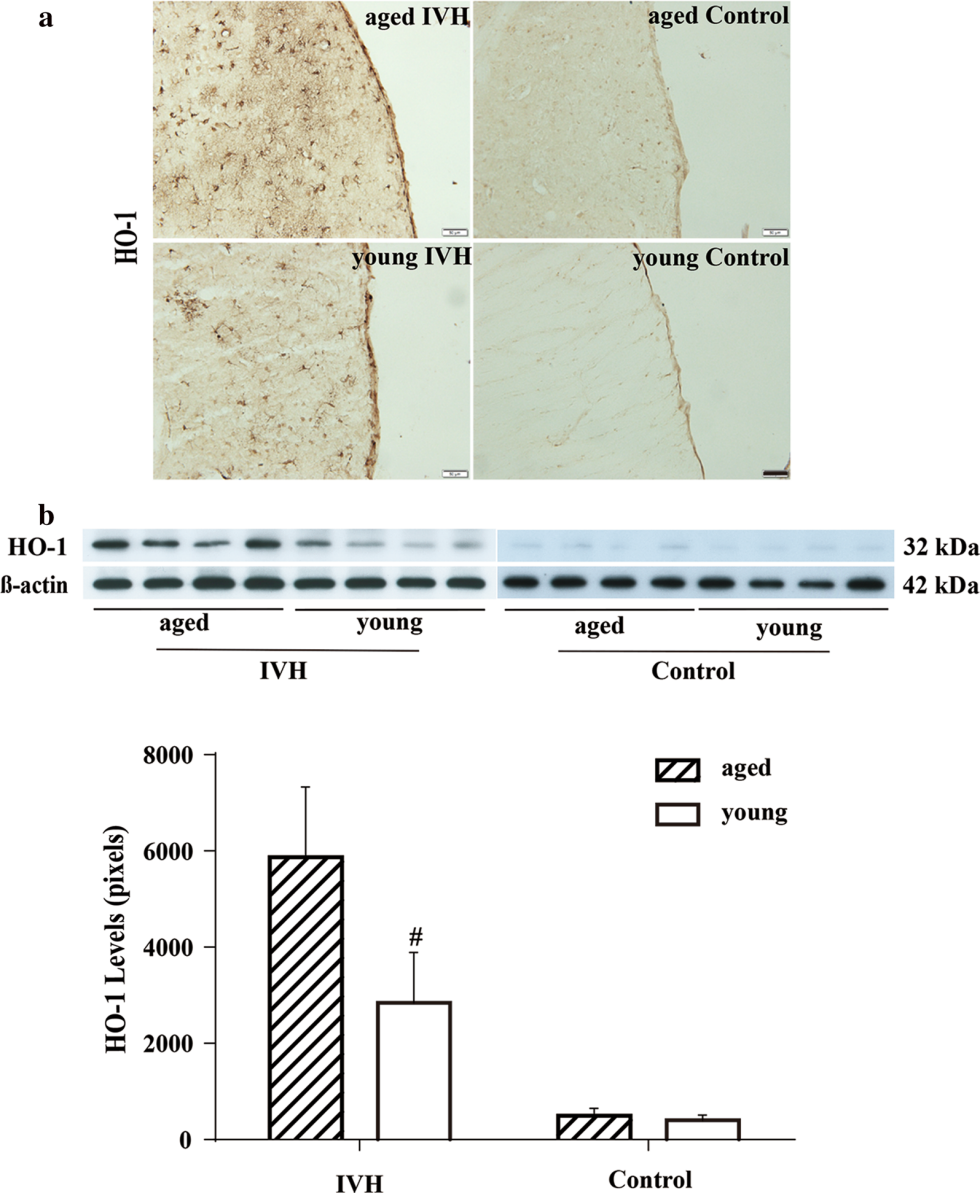
**a** Heme oxygenase (HO-1) immunoreactivity in the periventricular zone on day 1 after blood (200 µl) or saline injection into the right lateral ventricle in young (3 months) and aged (18 months) F344 rats. Scale bar = 50 μm. Note the increased HO-1 immunoreactivity after IVH in both young and aged rats compared to respective control rats, but the greater immunoreactivity in the aged rats. **b** Western blot of HO-1 in the periventricular area on day 1 after blood (200 µl) or saline injection in young and aged F344 rats with β-actin loading controls. HO-1 protein levels were quantified (bar graph). Values are mean ± SD, n = 4, #*p *< 0.01 vs. young IVH group by Student *t* test

To further investigate ventricular wall damaged after IVH, periventricular HO-1 expression was analyzed. Increased HO-1 positive cells were observed in periventricular area 1 day after IVH compared to saline injection (Fig. 3a). Meanwhile, Western blots of periventricular tissue showed an elevated level of HO-1 in aged rats compared to young rats on day 1 after IVH (5871 ± 1461 vs. 2840 ± 1052, p < 0.01, Fig. 3b). No difference was present between young and aged animals 1 day after saline injection (484 ± 194 vs. 377 ± 113 in young rats, p > 0.05, Fig. 3b).

### Iba-1 and CD68 positive macrophages in young and aged rats after IVH

**Fig. 4:**
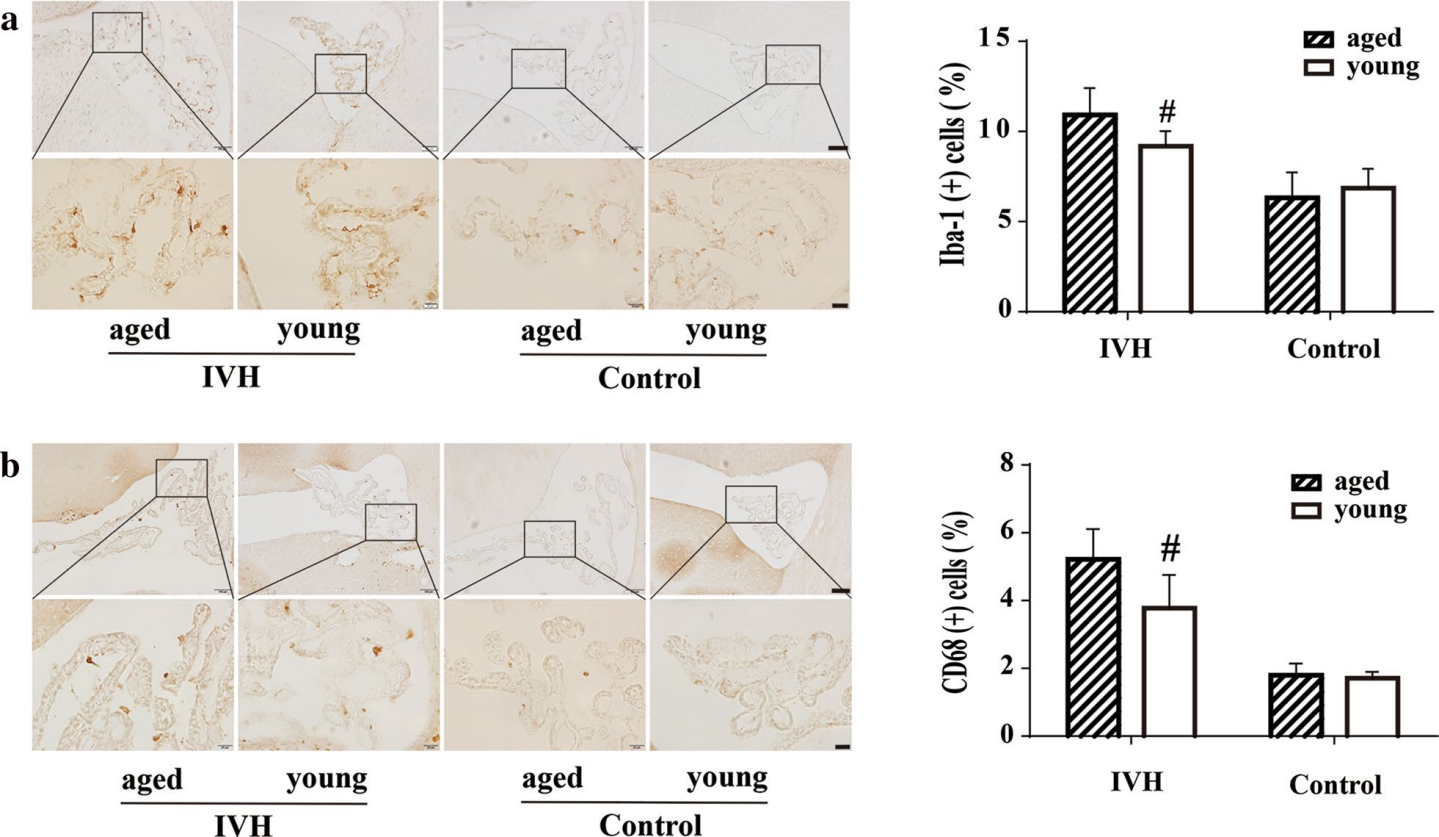
**a** Examples of Iba-1 immunoreactivity in macrophages of rats at 2 weeks in aged (18 months) IVH, young (3 months) IVH, aged control and young control groups. The number of Iba-1 positive cells was quantified relative to the number of choroid plexus epithelial cells. Values are mean ± SD; n = 13 in young and aged IVH groups and n = 6 in young and aged control groups. #*p *< 0.01 aged vs. young IVH groups by Student *t* test. Scale bar = 100 µm (upper row) and 20 µm (lower row). **b** Examples of CD68 immunoreactivity in macrophages of rats at 2 weeks in aged IVH, young IVH, aged control and young control groups. The number of CD68 positive cells was quantified relative to the number of choroid plexus epithelial cells. Values are mean ± SD; n = 13 in young and aged IVH groups and n = 6 in young and aged control groups. #*p *< 0.01 aged vs. young IVH groups by Student *t* test. Scale bar = 100 µm (upper row) and 20 µm (lower row)

Figure 4a showed an increase of choroid plexus Iba-1 positive macrophages on day 14 after IVH versus control group in both young and aged rats. The expression of Iba-1 in choroid plexus was significantly higher in aged IVH rats (10.9 ± 0.4% of all choroid plexus cells, n = 13) than that in young IVH rats (9.2 ± 0.2%, n = 13, p < 0.01, Fig. 4a). No difference was present between young and aged control groups (6.3 ± 0.7%; n = 6 vs. 6.8 ± 0.6%; n = 6 in young rats, p > 0.05, Fig. 4a).

A similar pattern was found in CD68 positive macrophages as shown in Fig. 4b. The expression of CD68 was increased on day 14 in IVH groups, and the expression of CD68 in choroid plexus was significantly higher in aged IVH rats (5.2 ± 0.2%, n = 13) compared with young IVH rats (3.8 ± 0.3%, n = 13, p < 0.01, Fig. 4b). No difference was present between young and aged control groups (1.8 ± 0.2%; n = 6 vs. 1.7 ± 0.1%; n = 6 in young rats, p > 0.05, Fig. 4b).

### Organized hematoma in the ventricle 14 days after IVH

**Fig. 5:**
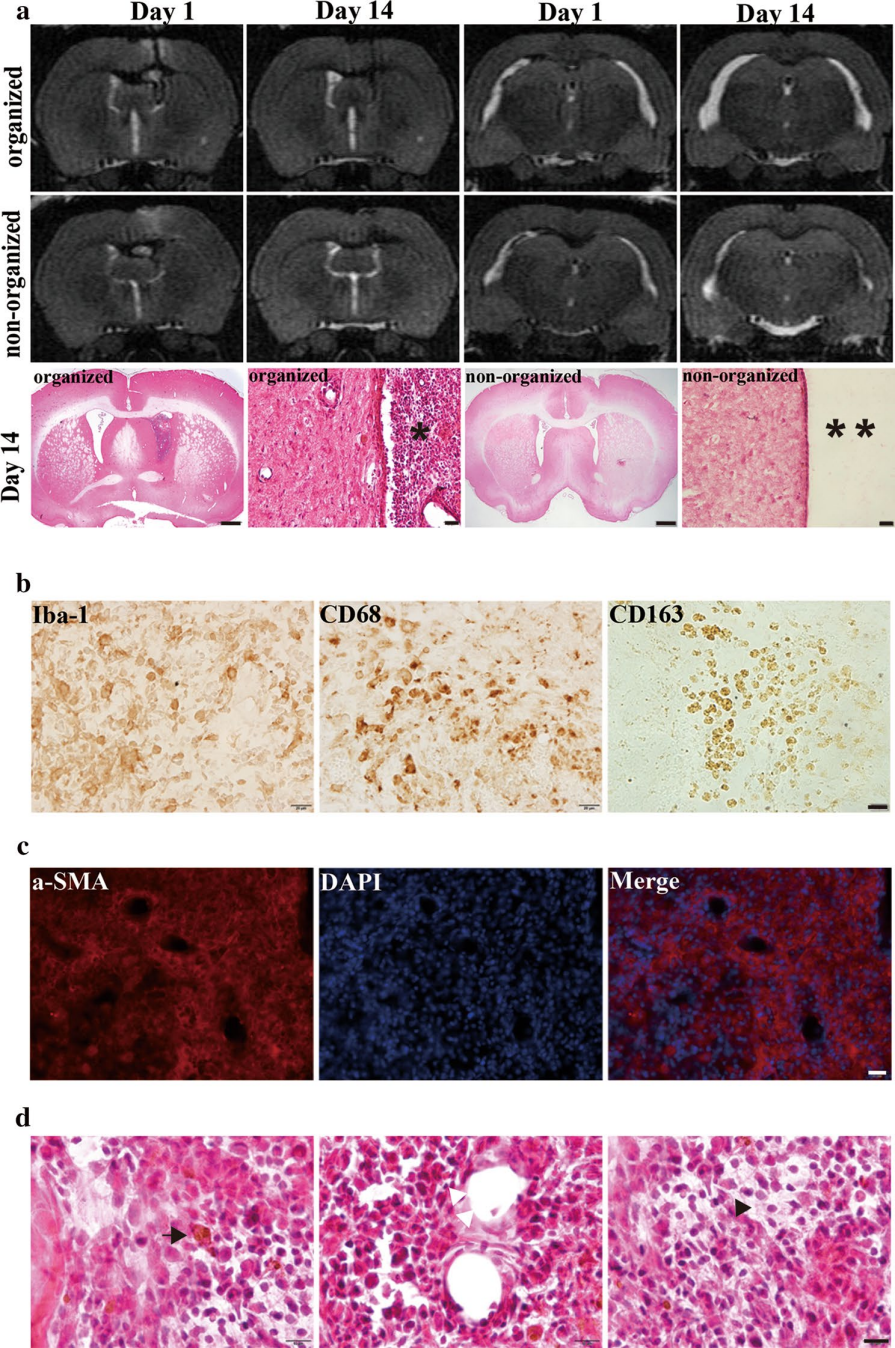
**a** Examples of T2-weighted MRIs at day 1 and 14 from two aged (18 months) F344 rats after intraventricular injection of blood (200 µl). In the top animal (organized hematoma), a clot remained in the right ventricle at day 14 and the ventricular system remained dilated. In the bottom animal (non-organized hematoma), the intraventricular clot resolved between day 1 and 14 and the ventricular dilatation reduced with time. Examination of the animals with H&E staining at day 14 revealed the presence of an organized clot (*) in the right ventricle in the first animal and no ventricular clot (**) in the second. Scale bar = 1 mm (left picture) and 20 mm (right picture). **b** Immunoreactivity for macrophage markers (Iba-1, CD68 and CD163) within the organized clot 2 weeks after IVH. Scale bar = 20 µm. **c** Immunofluorescence staining of alpha-smooth muscle actin (α-SMA) (fibrosis marker) at 2 weeks within the organized clot. Scale bar = 100 µm. **d** H&E staining showing a macrophage with hemosiderin (black arrow), neovascularization (white triangles) and hyalinization (black triangle) within the organized clot. Scale bar = 10 µm

Interestingly, organized intraventricular hematomas were observed in 3 of the aged rats 14 days after IVH (Fig. 5a), while the rest of the aged rats and all of the young rats presented with complete absorption of hematoma on day 14. The organized hematomas were immuno-positive for the microglia/macrophage markers Iba-1, CD68, and CD163 (Fig. 5b). Immunofluorescence of alpha-smooth muscle actin was also detected (Fig. 5c). H&E staining of the ventricle organized hematoma showed hemosiderin, macrophages, neovascularization and hyalinization (Fig. 5d).

### Ventricular enlargement and T2* lesions in organized hematoma

**Fig. 6:**
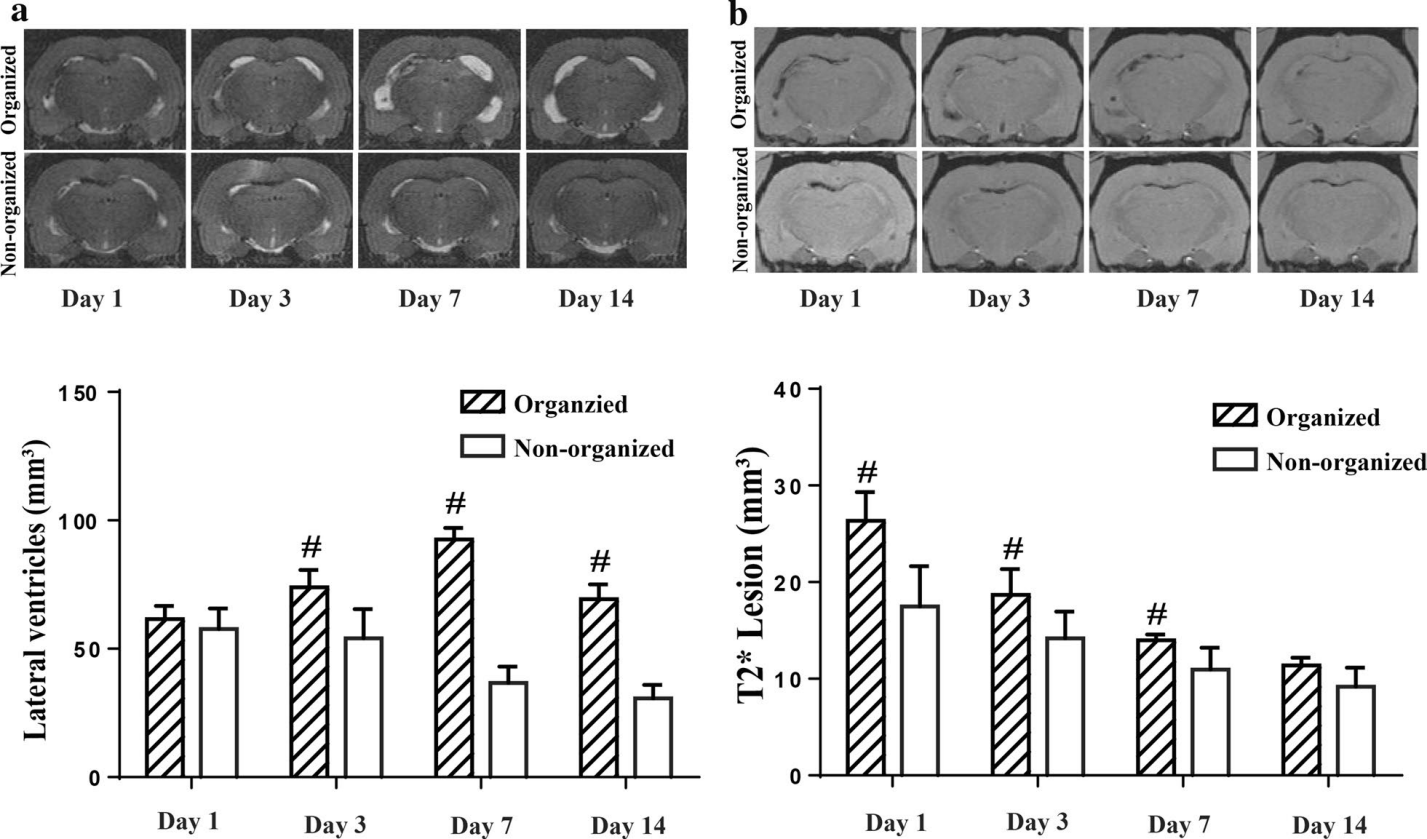
**a** Examples of coronal T2 images over 2 weeks in aged (18 months) rats that had an organized hematoma (organized) or did not (non-organized). Note the relative ventricular size. Ventricular volume was quantified in rats with an organized hematoma (n = 3) or did not (n = 10) from day-1 to -14 after blood injection. Values are expressed as the mean ± SD, #*p *< 0.01 vs. non-organized aged rats. **b** Examples of coronal T2* images for 2 weeks in aged rats that had an organized hematoma (organized) or did not (non-organized). Quantification of T2* lesions in the organized hematoma (n = 3) and non-organized hematoma (n = 10) aged rats from day-1 to -14 after blood injection. Values are expressed as the mean ± SD, #*p *< 0.01 vs. non-organized aged rats

Serial MRI in 77% (10/13) of aged IVH rats showed ventricular enlargement peaked on day 1 and gradually reduced over time. However, in 23% (3/13) of aged IVH rats, the ventricles gradually dilated and the ventricular volumes peaked on day 7 (Fig. 6a). Those aged IVH rats with progressive hydrocephalus were also found to have organized hematomas in the ventricles on day 14, therefore defined as organized hematoma (organized) rats, relative to the non-organized hematoma (non-organized) rats. The ventricular volumes in organized hematoma aged rats were significantly larger than those in the non-organized aged rats from day 3 to day 14 (p < 0.01, Fig. 6a).

To further explore the mechanisms of hydrocephalus development in organized aged rats, iron accumulation was examined using T2* weighted MRI after IVH. The volume of T2* lesions was larger in organized-hematoma IVH rats compared with the non-organized IVH rats from day 1 to day 7 (p < 0.01, Fig. 6b).

### Intraventricular iron injection in young and aged rats

**Fig. 7:**
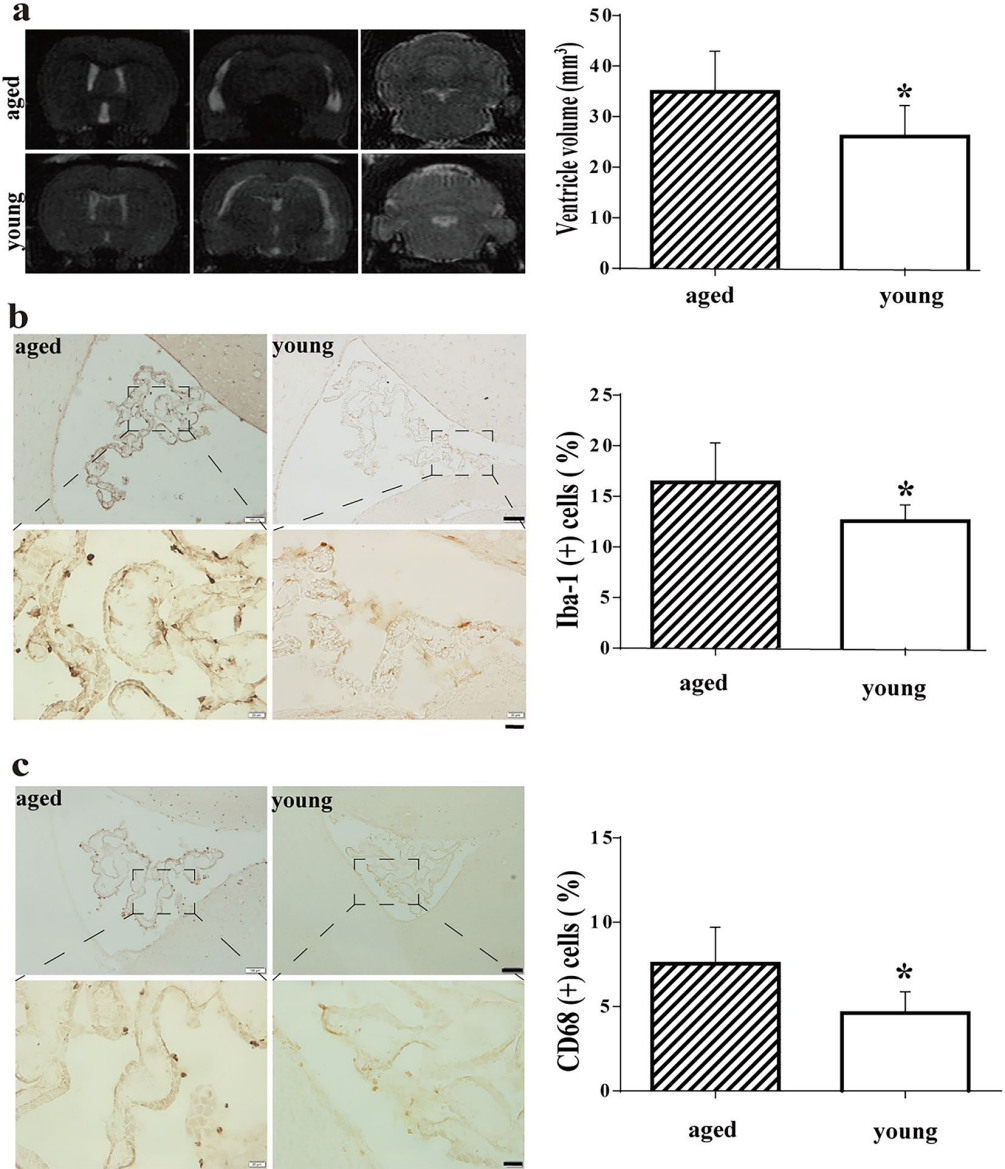
**a** Examples of T2-weighted MRIs 24 h after intraventricular injection of FeCl_3_ in young (3 months) and aged (18 months) rats. Note the bigger dilated ventricles in the aged rats. Ventricular volumes were quantified (bar graph). Values are mean ± SD; n = 7, *p < 0.05 vs. aged rats group by Student *t* test. **b** Iba-1 immunoreactivity in the choroid plexus 24 h after intraventricular injection of FeCl_3_ in young and aged rats. The number of Iba-1 positive cells was calculated and expressed relative to the number of choroid plexus epithelial cells. Values are mean ± SD; n = 7, *p < 0.05 vs. aged rats group by Student *t* test. Scale bar = 100 µm (upper and middle row) and 20 µm (lower row). (C) CD68 immunoreactivity in the choroid plexus 24 h after intraventricular injection of FeCl_3_ in young and aged rats. The number of Iba-1 positive cells was calculated and expressed relative to the number of choroid plexus epithelial cells. Values are mean ± SD; n = 7, *p < 0.05 vs. aged rats group by Student *t* test. Scale bar = 100 µm (upper and middle row) and 20 µm (lower row)

To further investigate the role of iron in hydrocephalus following IVH, 50 µl of iron (Fe^3+^) was injected into the right lateral ventricle of both young and aged rats. MRI showed that intraventricular iron injection induced greater intraventricular enlargement on day 1 in aged rats compared to young rats (34.9 ± 2.8 mm^3^; n = 7 vs. 26.1 ± 2.3 mm^3^ in the young rats; n = 7, p < 0.05, Fig. 7a). Additionally, aged rats had higher percentage of Iba-1 positive macrophages (16.4 ± 1.3% of choroidal epithelial cells; n = 7 vs. 12.5 ± 0.6% in young rats n = 7; p < 0.05, Fig. 7b) and CD68 positive macrophages (7.5 ± 0.8%, n = 7 vs. 4.6 ± 0.5% in the young rats, n = 7, p < 0.05, Fig. 7c).

## Discussion

This study used both young and aged rats to demonstrate the following findings. (1) Intraventricular injection of autologous arterial blood could induce hydrocephalus throughout day 1 to day 14 in both young and aged rats, while the aged rats developed more severe hydrocephalus than young rats through day 3 to day 14. (2) Both ependymal damage and periventricular HO-1 expression on day 1 after IVH were greater in aged compared to young rats. (3) Compared to young rats, aged rats had more prominent choroid plexus macrophage activation on day 14 after IVH. (4) Organized hemorrhages occurred in the brain ventricle of the some aged IVH rats. (5) Intraventricular iron injection may mimic the effect of IVH. Aged rats had more prominent hydrocephalus as well as an increased amount of Iba-1 and CD68 immuno-positive macrophages on day 1 after intraventricular iron injection compared with young rats.

Ependymal damage and disturbances in ventricular wall integrity may aggravate periventricular brain injury and hydrocephalus after IVH [5, 15]. Ependymal damage also leads to loss of cilia function. Absent or defective ependymal motile cilia has been postulated to have an important role in hydrocephalus development [16]. We observed greater ependymal damage in aged versus young IVH rats, which could contribute to the accentuated hydrocephalus in aged IVH rats.

HO-1 (also known as heat shock protein 32) is a key enzyme in heme degradation, upregulated primarily in microglia after cerebral hemorrhage. HO-1 may be partly responsible for brain iron overload and subsequent brain damage after ICH and subarachnoid hemorrhage [6, 11]. Studies have established HO-1 inhibitors treatment could decrease ICH or intracerebral hemoglobin induced brain edema studies have proved HO-1 inhibitors treatment could decrease brain edema after ICH or intracerebral hemoglobin injection [17, 18]. Therefore, a higher expression of HO-1 in aged IVH rats may be responsible for advanced hydrocephalus. The greater HO-1 upregulation in the aged animals may also reflect greater ependymal damage which may affect how far hemoglobin and iron penetrates into the periventricular zone.

Some recent studies indicate that inflammatory activation of the choroid plexus may trigger hydrocephalus development after IVH [9]. Epiplexus cells, also known as “Kolmer cells”, are found on the apical surface of the choroid plexus facing the CSF [19]. Functions include the production and release of nitric oxide, antigen presentation, phagocytosis and clearance of foreign bodies. Hence they are considered to have an immunological role as macrophages in the brain ventricles [20]. Our previous study found that spontaneously hypertensive rats exhibit epiplexus cell activation during the development of hydrocephalus [10] and such activation was also associated with hydrocephalus after subarachnoid and IVH [21]. Iba-1 and CD68 are microglia/macrophage markers in rat brain [22, 23] and, in the current study, aged rats had a higher choroid plexus expression of both markers compared to young rats after IVH. This difference in choroid plexus macrophag**e** activation in aged animals correlated with a greater degree of hydrocephalus in those animals suggesting a potential link between choroid plexus inflammatory events and hydrocephalus.

Unexpectedly, we found organized hematomas in the ventricles in a subgroup of aged rats 2 weeks after intraventricular injection of 200 µl autologous arterial blood. Reports have described organized hematomas in other parts of the brain, including maxillary sinuses [24] and subdurally [25]. It should be noted that organized hematomas do not occur in intracerebral hemorrhage. The organized hematomas after IVH were positive for a microglia/macrophage marker (Iba-1), macrophage phenotype markers (CD68, CD163), and a fibrosis marker (alpha-smooth muscle actin). In addition, hemosiderin-laden macrophages, neovascularization, and hyalinization were seen in the ventricular organized hematomas with high magnification H&E staining.

In the clinical setting, external ventricular drains (EVD) are placed in some patients to alleviate hydrocephalus symptoms in the initial phase after IVH [7], but the mechanisms of hydrocephalus development after IVH are still not fully understood. The current study found that IVH-induced ventricular dilatation typically peaked on day 1 after hemorrhage and gradually decreased in all young rats and most (10/13) aged IVH rats. However, 23% (3/13) of aged IVH rats showed progression of hydrocephalus and the ventricular dilatation peaked on day 7 after IVH. This phenomenon of progressive hydrocephalus may be related to the prominent iron deposition observed in T2* MRI from day 1 in those animals. Our recent study suggested that iron could play a critical role in hydrocephalus development after IVH [5]. While the recent CLEAR III trial using tissue-type plasminogen activator (alteplase) with an EVD to accelerate hematoma clearance in adult IVH patients with hydrocephalus showed an improvement in survival, it did not improve functional outcome [26]. There has, therefore, been interest in developing ways of stratifying patients who might benefit from that intervention [27]. It is possible that T2* MRI might be a stratification tool.

After IVH, blood in the CSF and extracellular fluid increases the resistance to CSF drainage and then causes inflammatory response with an arachnoiditis. Iron release after red blood cell lysis and hemoglobin degradation plays a critical part in brain injury [11, 28]. Iron levels in CSF are significantly elevated after IVH [29] and the current study shows that intraventricular injection of iron can mimic the acute ventricle enlargement and epiplexus cell activation found after IVH. As with IVH, those effects were exacerbated by aging suggesting that iron might play a role in age-dependent hydrocephalus development after IVH.

In the current study, we demonstrated that the effects of aging on hydrocephalus after IVH, although effects of aging on ependymal injury and choroid plexus macrophage activation were moderate. However, there are several limitations in this study. (1) Only male rats were used and sex differences were not studied; (2) It is well known that hydrocephalus causes cognitive deficits, however, functional outcomes were not measured; (3) The iron injection model was only used as a proof-of-concept study. Although both iron and IVH caused hydrocephalus, the acidic iron solution (pH 3.0) makes it difficult to compare quantitatively to the IVH model. We have previously shown that systemic deferoxamine, an iron chelator, can reduce IVH-induced hydrocephalus in young rats [5]; and (4) Whether clot removal can reduce hydrocephalus and brain injury after IVH was not examined. A large IVH animal model needs to be established to test this hypothesis.

## Conclusions

In conclusion, age impacts hydrocephalus development after IVH results with more severe hydrocephalus in aged rats. This was associated with increased ventricular wall ependymal damage and more inflammation (Iba-1 and CD68 positive macrophages) at the choroid plexus. The hydrocephalus induced by intraventricular injection of iron was also more severe in aged animals as was the periventricular induction of HO-1 after IVH suggesting a difference in heme/iron handling with age. Determining the mechanism underlying age-related exacerbation of hydrocephalus after IVH could lead to potential therapeutic targets in the elderly population.

## Data Availability

The datasets used and/or analyzed during the current study are available from the corresponding author on reasonable request.

## Abbreviations

CSF: cerebrospinal fluid
EVD: external ventricular drains
HO-1: heme oxygenase-1
H&E: hematoxylin and eosin
ICH: intracerebral hemorrhage
IVH: intraventricular hemorrhage
MRI: magnetic resonance imaging
SD: standard deviation
