# Correction: Effects of aging on hydrocephalus after intraventricular hemorrhage

**DOI:** 10.1186/s12987-024-00591-z

**Published:** 2024-12-03

**Authors:** Yingfeng Wan, Feng Gao, Fenghui Ye, Weiming Yang, Ya Hua, Richard F. Keep, Guohua Xi

**Affiliations:** 1 Department of Neurosurgery, University of Michigan, R5018 Biomedical Science Research Building, 109 Zina Pitcher Place, Ann Arbor, MI 48109‑2200 USA; 2 Department of Neurosurgery, Sir Run Run Shaw Hospital, Zhejiang University, Hangzhou, China; 3 Department of Neurology, 2nd Affiliated Hospital, Zhejiang University, Hangzhou, China

**Correction: Fluids Barriers CNS17, 8 (2020)**

10.1186/s12987-020-0169-y

In the original publication of this article, a mistake was made in creating Fig. 7A. The two examples of MRIs in the 3rd column showing the hindbrain (outlined in red) were incorrect. This is corrected in the new figure supplied below. This mistake does not change any of the analyses in the paper. All the ventricular measurements in the study were of the lateral ventricles and do not involve the hindbrain.


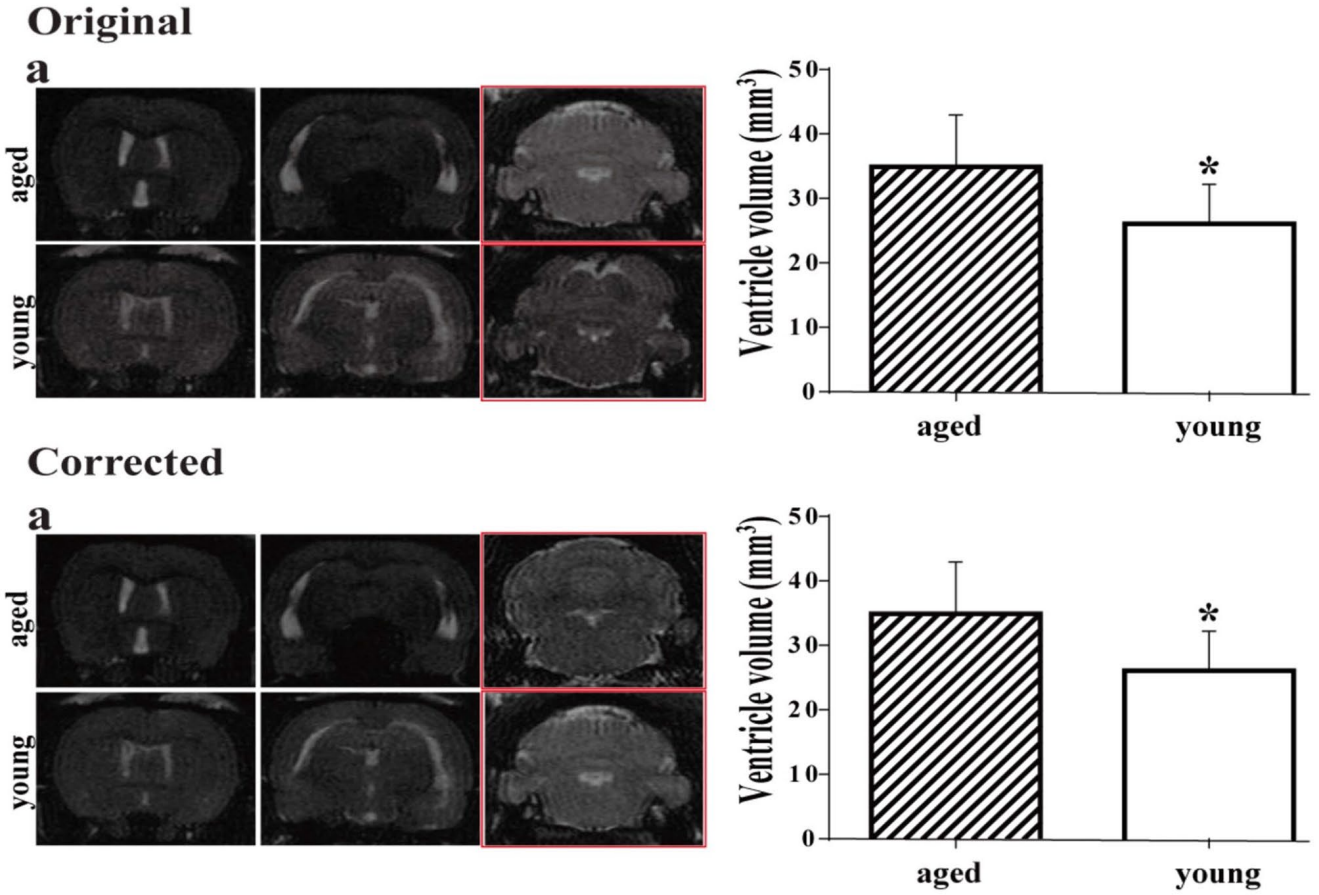


The original article has been corrected.

